# Automated Electronic Alert for the Care and Outcomes of Adults With Acute Kidney Injury: A Randomized Clinical Trial

**DOI:** 10.1001/jamanetworkopen.2023.51710

**Published:** 2024-01-19

**Authors:** Ting Li, Buyun Wu, Li Li, Ao Bian, Juan Ni, Kang Liu, Zhongke Qin, Yudie Peng, Yining Shen, Mengru Lv, Xinyi Lu, Changying Xing, Huijuan Mao

**Affiliations:** 1 Department of Nephrology, Jiangsu Province Hospital, The First Affiliated Hospital of Nanjing Medical University, Nanjing, China

## Abstract

**Importance:**

Despite the expansion of published electronic alerts for acute kidney injury (AKI), there are still concerns regarding their effect on the clinical outcomes of patients.

**Objective:**

To evaluate the effect of the AKI alert combined with a care bundle on the care and clinical outcomes of patients with hospital-acquired AKI.

**Design, Setting, and Participants:**

This single-center, double-blind, parallel-group randomized clinical trial was conducted in a tertiary teaching hospital in Nanjing, China, from August 1, 2019, to December 31, 2021. The inclusion criteria were inpatient adults aged 18 years or older with AKI, which was defined using the Kidney Disease: Improving Global Outcomes creatinine criteria. Participants were randomized 1:1 to either the alert group or the usual care group, which were stratified by medical vs surgical ward and by intensive care unit (ICU) vs non-ICU setting. Analyses were conducted on the modified intention-to-treat population.

**Interventions:**

A programmatic AKI alert system generated randomization automatically and sent messages to the mobile telephones of clinicians (alert group) or did not send messages (usual care group). A care bundle accompanied the AKI alert and consisted of general, nonindividualized, and nonmandatory AKI management measures.

**Main Outcomes and Measures:**

The primary outcome was maximum change in estimated glomerular filtration rate (eGFR) within 7 days after randomization. Secondary patient-centered outcomes included death, dialysis, AKI progression, and AKI recovery. Care-centered outcomes included diagnostic and therapeutic interventions for AKI.

**Results:**

A total of 2208 patients (median [IQR] age, 65 [54-72] years; 1560 males [70.7%]) were randomized to the alert group (n = 1123) or the usual care group (n = 1085) and analyzed. Within 7 days of randomization, median (IQR) maximum absolute changes in eGFR were 3.7 (−6.4 to 19.3) mL/min/1.73 m^2^ in the alert group and 2.9 (−9.2 to 16.9) mL/min/1.73 m^2^ in the usual care group (*P* = .24). This result was robust in all subgroups in an exploratory analysis. For care-centered outcomes, patients in the alert group had more intravenous fluids (927 [82.6%] vs 670 [61.8%]; *P* < .001), less exposure to nonsteroidal anti-inflammatory drugs (56 [5.0%] vs 119 [11.0%]; *P* < .001), and more AKI documentation at discharge (560 [49.9%] vs 296 [27.3%]; *P* < .001) than patients in the usual care group. No differences were observed in patient-centered secondary outcomes between the 2 groups.

**Conclusions and Relevance:**

Results of this randomized clinical trial showed that the electronic AKI alert did not improve kidney function or other patient-centered outcomes but changed patient care behaviors. The findings warrant the use of a combination of high-quality interventions and AKI alert in future clinical practice.

**Trial Registration:**

ClinicalTrials.gov Identifier: NCT03736304

## Key Points

**Question:**

Can an acute kidney injury (AKI) alert combined with a care bundle improve the care and clinical outcomes of hospitalized patients with AKI?

**Findings:**

In this randomized clinical trial involving 2208 adults with hospital-acquired AKI, the AKI alert did not improve short-term kidney function and other clinical outcomes compared with usual care. It prompted changes in patient care of AKI, increasing intervention rates and AKI diagnosis, but these changes did not contribute to favorable outcomes.

**Meaning:**

Findings of this trial warrant the use of a combination of AKI alert and high-quality interventions in future clinical practice.

## Teaser

This randomized clinical trial evaluates the implementation of an automated warning system and its effect on the management and outcomes of acute kidney injury (AKI).

## Introduction

Acute kidney injury (AKI) is a common and serious complication in hospitalized patients, affecting up to 18% of general inpatients and greater than 50% of patients in the intensive care unit (ICU).^1^ The occurrence of AKI increases the risk of in-hospital mortality and the development of cardiovascular disease and chronic kidney disease, with substantial resource and economic implications.^2,3,4^ Early detection and prompt intervention are critical for improved patient outcomes.

The electronic AKI alert is a computerized algorithm that uses changes in serum creatinine (SCr) to identify AKI early. The AKI alert notifies clinicians of an AKI episode, allowing for prompt interventions. However, 10 nonrandomized clinical trials and 3 randomized clinical trials (RCTs) of the alert system had mixed results. Seven of 10 nonrandomized clinical trials reported improved patient-centered outcomes, including improved AKI recovery,^5,6,7^ reduced mortality and dialysis rates,^8,9,10^ and decreased hospital length of stay (LOS).^9,11,12^ In contrast, RCT results have suggested that AKI alerts were less beneficial, possibly even causing harm in specific instances.^13,14,15^ A 2015 trial by Wilson et al^13^ showed increased dialysis rates in the surgical ward subgroup. In 2021, another trial revealed higher death rates in nonteaching hospitals from AKI alerts compared with usual care.^14^ These inconsistent results might be explained by the diversity in patient population, timeliness of AKI alerts, hierarchy of disruption, and alert time and content, each of which warrants further investigation.^16^

Medical education and practice patterns vary by different regions in the world. It is unknown whether the AKI alert has benefits for hospitalized patients in China, in which specialized physicians receive less nephrology training. Therefore, we conducted this trial to assess the effect of the AKI alert combined with a care bundle on the care and clinical outcomes of patients with hospital-acquired AKI.

## Methods

### Trial Design

This single-center, double-blind, parallel-group RCT was conducted from August 1, 2019, to December 31, 2021, in a tertiary teaching hospital in Nanjing, China. The Nanjing Medical University Institutional Review Board approved the trial and waived the informed consent requirement because the study posed minimal risk and contacting participants for consent would be an intervention itself and might have affected the results. The trial was conducted according to the Declaration of Helsinki^17^; the trial protocol is provided in Supplement 1. We followed the Consolidated Standards of Reporting Trials (CONSORT) reporting guideline.

### Participants

The inclusion criteria were inpatient adults 18 years or older with AKI. All patients were Han Chinese. Acute kidney injury was defined according to the Kidney Disease: Improving Global Outcomes (KDIGO) creatinine criteria: an increase in SCr of at least 0.3 mg/dL (to convert SCr to micromoles per liter, multiply by 88.4) within 48 hours or by 1.5 times the lowest measured SCr within the previous 7 days. The specific KDIGO AKI stages are as follows: stage 1, a 1.5- to 1.9-times increase in SCr from baseline or 0.3-mg/dL or higher increase in SCr within 48 hours; stage 2, a 2.0- to 2.9-times increase in SCr from baseline; and stage 3, a 3.0-times or higher increase in SCr from baseline, or increase in SCr of 4.0 mg/dL or higher, or initiation of kidney replacement therapy. Exclusion criteria were baseline estimated glomerular filtration rate (eGFR) lower than 15 mL/min/1.73m^2^, admission diagnosis of end-stage kidney disease, history of kidney transplant, AKI occurring outside the hospital, hospitalization for less than 24 hours, and baseline SCr levels lower than 0.5 mg/dL. The eGFR was calculated using the Chronic Kidney Disease Epidemiology Collaboration equation. For patients with recurring hospitalizations, only the first admission during the study period was used.

### Intervention

The AKI alert was generated by comparing real-time SCr test results with historic SCr measurements. The alert system generated randomization automatically and sent messages to the mobile telephones of clinicians (alert group) or did not send messages (usual care group). The message included an AKI alert and a care bundle. The care bundle comprised general, nonindividualized, and nonmandatory AKI management measures. If a patient triggered multiple alerts of AKI, the message was sent up to a maximum of 3 times to prevent alert fatigue.

The content of the message was as follows: “Greetings, the patient in bed [number], in the [ward name] ward, admitted on [date], has a serum creatinine result of [SCr value] from the [date] test time. Based on the creatinine result, acute kidney injury is probable to occur. Acute kidney injury requires optimization of hemodynamics, discontinuation of unnecessary nephrotoxic drugs, and adjustment of antimicrobial drug dosage and dialysis if necessary. Please be vigilant and handle it accordingly. Thank you! For diagnosis and treatment inquiries, please contact the nephrology consultation service at the kidney consultation phone number [number].”

### Outcomes

The primary patient-centered outcome was a maximum change in eGFR within 7 days after randomization. The maximum absolute change in eGFR within 7 days was the eGFR at AKI minus the lowest eGFR within 7 days of randomization. If the patient died, eGFR was considered to be equal to 0. The maximum relative change in eGFR was the calculated difference (eGFR at AKI minus lowest eGFR) divided by the eGFR at randomization.

Secondary patient-centered outcomes were maximum change in SCr level within 7 days, dialysis within 7 days, death within 7 days, in-hospital death, in-hospital dialysis, death within 28 days, death within 90 days, progression of AKI, highest AKI stage achieved, AKI recovery at discharge, and incidence of being alive with dialysis dependency at 90 days. Prespecified care-centered outcomes included intravenous fluids, urinalysis, fluid intake and output measurement, SCr level measurement, kidney ultrasonography, correcting anemia by reaching hemoglobin level over 9.0 g/dL (to convert to grams per liter, multiply by 10.0), and nephrologist consultation within 2 days after randomization as well as discontinuation of nephrotoxic medications (including contrast, aminoglycoside, vancomycin, chemotherapy, nonsteroidal anti-inflammatory drugs, and angiotensin-converting enzyme inhibitors and angiotensin receptor blockers) within 3 and 7 days after randomization.

### Sample Size Calculation

On the basis of pilot data, we estimated an SD of 13.4 mL/min/1.73m^2^ of absolute eGFR change averaged across study groups. We considered a relative 10% reduction in the primary outcome (from 19.0 to 17.1 mL/min/1.73m^2^) to be clinically significant. At a 2-tailed .025-level test for an overall significance level of α = .05, a sample size of 1050 in each group of the study was deemed sufficient to achieve 90% power to detect the difference. Given the potential for 30% of patients without a follow-up SCr level measurement, the sample size was inflated to 3000 cases.

### Randomization and Masking

**Figure 1.**
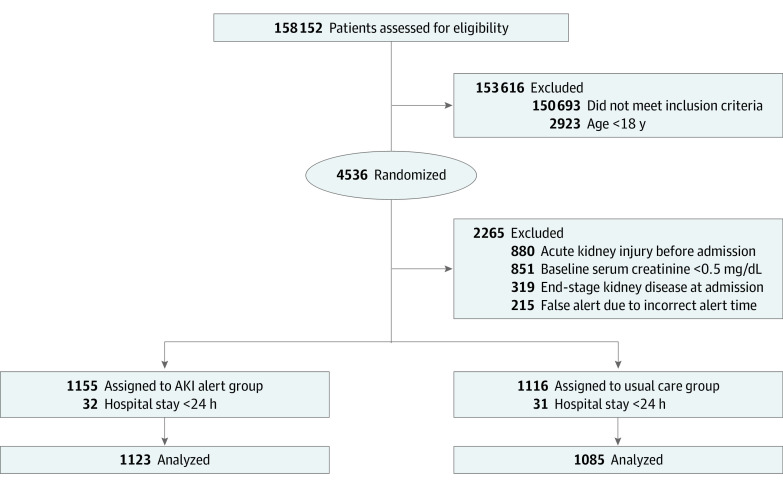
Trial Flowchart AKI indicates acute kidney injury. To convert creatinine to micromoles per liter, multiply by 88.4.

Eligible patients were randomized 1:1 to either the alert group or the usual care group (Figure 1). Computer-based randomization was performed by a statistician independent of the trial analysis. Based on the a priori hypothesis that the type of service and location could influence the effect of the AKI alert, as also suggested by Wilson et al,^13^ we divided patients into 4 groups based on the combination of medical vs surgical ward and ICU vs non-ICU setting. Each group was subsequently randomized using the universal seed number 20180101. The specific seed values used within each stratum were the consecutive increments of the initial value. Data were collected in person from electronic medical records at baseline and for follow-up. All study investigators and participants were blinded to patient randomization status, although clinicians were aware of the intervention measures in the alert group. Investigators did not review the data until the data were unmasked.

### Statistical Analysis

Baseline characteristics were summarized and presented according to study groups. Continuous variables were presented as medians with IQRs, and categorical variables were expressed as absolute numbers and percentages. Differences in variables were compared using the Mann-Whitney test and χ^2^ test as appropriate. Analyses were conducted on the modified intention-to-treat population, which consisted of randomized participants who experienced hospital-acquired AKI, received timely alert notifications, had an inpatient stay exceeding 24 hours, and had a baseline SCr level over 0.5 mg/dL. We performed exploratory subgroup analyses by sex, age, hypertension, diabetes, baseline eGFR, AKI stage, and wards at randomization for the primary and main secondary outcomes, applying no correction for multiple testing of exploratory outcomes. Univariate binary logistic regression was used to assess the effectiveness of the AKI alert across subgroups, and interaction terms were included as modifiers of the main effect.

Two-sided *P* < .05 was considered to be statistically significant. No imputation for missing data was performed. Statistical analyses were performed using IBM SPSS Statistics, version 25 (IBM Corp). Data were graphically displayed using R, version 4.3.0 (R Project for Statistical Computing).

## Results

### Participant Characteristics

During the study period, there were a total of 158 152 hospitalized adults assessed for eligibility, of whom 4536 triggered AKI alerts and were randomized. Due to the inability of the alert system to precisely exclude all patients who did not meet the inclusion criteria, a larger number of patients than expected were excluded during the data analysis phase. As a result, 2208 patients were identified as having hospital-acquired AKI, giving an overall incidence of 1.4 AKI episodes per 100 admissions. At randomization, 1123 patients were assigned to the alert group and 1085 patients were assigned to the usual care group (Figure 1).

The 2208 patients in the modified intention-to-treat analysis had a median (IQR) age of 65 (54-72) years and included 1560 males (70.7%) and 648 females (29.3%). At the time of randomization, 1338 patients (60.6%) were in the ICU. The median (IQR) LOS from admission to randomization was 7 (2-13) days. Seven cases (0.6%) in the usual care group and 14 cases (1.2%) in the alert group lacked complete blood count data, while 39 cases (3.6%) in the usual care group and 60 cases (5.3%) in the alert group had missing electrolyte results. Primary and secondary outcome data were complete. Demographic and clinical characteristics were similar between the study groups (Table 1).

**Table 1.** Patient Characteristics at Time of Randomization

Characteristic	Patients, No. (%)	
	Usual care group (n = 1085)	Alert group (n = 1123)
Demographics		
Age, median (IQR), y	65 (55-73)	64 (54-72)
Sex		
Male	784 (72.3)	776 (69.1)
Female	301 (27.7)	347 (30.9)
Current smoker status	269 (24.8)	277 (24.7)
Current alcohol use	134 (12.4)	157 (13.9)
Hospital admission		
Patient in ICU	637 (58.7)	701 (62.4)
Patient in ward	448 (41.3)	422 (37.6)
Comorbidities		
CKD	250 (23.0)	237 (21.1)
CHF	212 (19.5)	226 (20.1)
COPD	38 (3.5)	44 (3.9)
Cerebrovascular disease	217 (20.0)	205 (18.3)
Diabetes	219 (20.2)	256 (22.8)
Hypertension	568 (52.4)	586 (52.2)
Malignant neoplasm	229 (21.1)	218 (19.4)
Liver disease	83 (7.7)	107 (9.5)
Laboratory values, median (IQR)^a^		
Creatinine at AKI, mg/dL	1.54 (1.17-2.08)	1.54 (1.20-2.01)
eGFR at AKI, mL/min/1.73m^2^	44.2 (30.2-60.0)	43.6 (31.1-58.5)
Baseline creatinine, mg/dL^b^	0.87 (0.70-1.18)	0.87 (0.70-1.17)
Urea nitrogen, mg/dL	22.5 (16.6-32.5)	21.5 (16.0-31.3)
WBC counts, ×10^3^/μL	7.96 (5.59-11.94)	7.72 (5.52-11.74)
Hemoglobin, g/dL	12.4 (10.4-14.0)	12.6 (10.5-14.2)
Platelet counts, ×10^3^/μL	166 (117-218)	171 (125-224)
Sodium, mEq/L	141 (138-143)	141 (138-143)
Potassium, mEq/L	3.84 (3.53-4.16)	3.84 (3.53-4.19)
Chloride, mEq/L	105 (102-108)	105 (102-108)
Calcium, mg/dL	8.76 (8.24-9.12)	8.76 (8.28-9.16)
Phosphorus, mg/dL	3.31 (2.69-3.90)	3.31 (2.66-3.90)
Time from admission to AKI, median (IQR), d	6.0 (2.0-12.0)	7.0 (2.0-13.0)

Abbreviations: AKI, acute kidney injury; CHF, congestive heart failure; CKD, chronic kidney disease; COPD, chronic obstructive pulmonary disease; eGFR, estimated glomerular filtration rate; ICU, intensive care unit; WBC, white blood cell.

SI conversion factors: To convert calcium to millimoles per liter, multiply by 0.25; chloride to millimoles per liter, multiply by 1.0; creatinine to micromoles per liter, multiply by 88.4; hemoglobin to grams per liter, multiply by 10.0; platelet count to ×10^9^ per liter, multiply by 1.0; phosphorus to millimoles per liter, multiply by 0.323; potassium to millimoles per liter, multiply by 1.0; sodium to millimoles per liter, multiply by 1.0; urea nitrogen to millimoles per liter, multiply by 0.357; and WBC to ×10^9^ per liter, multiply by 0.001.

^a^ Values at randomization unless otherwise specified.

^b^ Values in the 7 days before randomization.

### Primary, Secondary, and Patient Care Outcomes

Regarding the primary outcome, the median (IQR) maximum absolute changes in eGFR within 7 days after randomization were 3.7 (−6.4 to 19.3) mL/min/1.73 m^2^ in the alert group and 2.9 (−9.2 to 16.9) mL/min/1.73 m^2^ in the usual care group (*P* = .24) (Table 2). There was also no difference in the median (IQR) maximum relative changes in eGFR within 7 days after randomization between the alert and usual care groups (9.4% [−14.8% to 47.0%] vs 7.7% [−18.6% to 44.0%]; *P* = .26).

**Table 2.** Primary and Secondary Outcomes

Outcome	Patients, No. (%)		Difference (95% CI)	*P* value
	Usual care group (n = 1085)	Alert group (n = 1123)		
Primary outcomes, median (IQR)				
Maximum absolute changes in eGFR within 7 d after randomization, mL/min/1.73m^2^	2.9 (−9.2 to 16.9)	3.7 (−6.4 to 19.3)	1.1 (−0.8 to 3.0)	.24
Maximum relative changes in eGFR within 7 d after randomization, %	7.7 (−18.6 to 44.0)	9.4 (−14.8 to 47.0)	1.2 (−0.4 to 5.9)	.26
Maximum absolute changes in eGFR within 7 d after randomization after excluding deceased patients, mL/min/1.73m^2^	0.6 (−10.9 to 10.6)	0.9 (−9.0 to 11.6)	0.9 (−0.8 to 2.5)	.31
Maximum relative changes in eGFR within 7 d after randomization after excluding deceased patients, %	1.6 (−23.0 to 28.4)	2.4 (−19.9 to 29.6)	1.5 (−2.2 to 5.3)	.43
Secondary outcomes				
Maximum absolute changes in creatinine within 7 d after randomization after excluding deceased patients, median (IQR), mg/dL	0.0 (−0.5 to 0.2)	0.0 (−0.5 to 0.2)	0.0 (0.0 to 0.1)	.59
Maximum relative changes in creatinine within 7 d after randomization after excluding deceased patients, median (IQR), %	−0.7 (−31.8 to 17.1)	−1.9 (−33.5 to 15.0)	−1.4 (−4.8 to 1.8)	.40
Death within 7 d after randomization	112 (10.3)	134 (11.9)	1.6 (−1.0 to 4.2)	.23
In-hospital death	192 (17.7)	223 (19.9)	2.2 (−1.1 to 5.4)	.19
Death within 28 d after randomization	369 (34.0)	400 (35.6)	1.6 (−2.4 to 5.6)	.43
Death within 90 d after randomization	407 (37.5)	429 (38.2)	0.7 (−3.3 to 4.7)	.74
Dialysis within 7 d after randomization	118 (10.9)	148 (13.2)	2.3 (−0.4 to 5.0)	.10
In-hospital dialysis	172 (15.9)	211 (18.8)	2.9 (−0.2 to 6.1)	.07
In-hospital AKI progression	291 (30.2)	284 (28.7)	−1.5 (−5.6 to 2.5)	.41
Stage 1 to 2	137 (17.4)	130 (16.5)	−0.9 (−4.6 to 2.8)	.63
Stage 1 to 3	108 (13.7)	109 (13.8)	0.1 (−3.3 to 3.5)	.95
Stage 2 to 3	46 (26.2)	45 (22.4)	−3.8 (−12.4 to 4.9)	.38
Highest AKI stage achieved				
1	548 (50.5)	556 (49.5)	−1 (−5.2 to 3.2)	.73
2	269 (24.8)	295 (26.3)	1.5 (−2.2 to 5.1)	
3	268 (24.7)	272 (24.2)	−0.5 (−4.1 to 3.1)	
AKI recovery at discharge	566 (63.4)	595 (60.2)	−3.2 (−7.5 to 1.2)	.70
Incidence of being alive with dialysis dependency at 90 d	54 (7.9)	43 (6.4)	−1.5 (−4.4 to 1.4)	.19

Abbreviations: AKI, acute kidney injury; eGFR, estimated glomerular filtration rate.

SI conversion factor: To convert creatinine to micromoles per liter, multiply by 88.4.

Regarding the secondary outcomes, there were no significant differences between the 2 groups in the median (IQR) maximum absolute changes in SCr level (0.0 [−0.5 to 0.2] mg/dL for both groups; *P* = .59) and maximum relative changes in SCr level (−1.9% [−33.5% to 15.0%] vs −0.7% [−31.8% to 17.1%]; *P* = .40) within 7 days after randomization. Similarly, there were no statistically significant differences between the 2 groups in other patient-centered secondary outcomes, including in-hospital death, in-hospital dialysis, dialysis within 7 days, death within 7 days, death within 28 days, death within 90 days, in-hospital AKI progression, highest AKI stage achieved, AKI recovery at discharge, and incidence of being alive with dialysis dependency at 90 days (Table 2).

The effect of AKI alerts on patient care outcomes is shown in Table 3. Within 2 days after randomization, there was a significantly higher number of patients receiving intravenous fluids in the alert group compared with the usual care group (927 patients [82.6%] vs 670 patients [61.8%]; *P* < .001). We also observed a higher number of patients with urinalysis (407 [36.2%] vs 141 [13.0%]; *P* < .001), fluid intake and output measurements (995 [88.6%] vs 743 [68.5%]; *P* < .001), subsequent SCr level measurement (1039 [92.5%] vs 814 [75.0%]; *P* < .001), kidney ultrasonography (59 [5.3%] vs 6 [0.6%]; *P* < .001), and correcting anemia by reaching hemoglobin level over 9.0 g/dL (517 [46.0%] vs 252 [23.2%]; *P* < .001) in the alert group compared with the usual care group. No significant difference was found between the groups in terms of nephrologist consultation rates overall. More patients in the usual care group than in the alert group were exposed to nonsteroidal anti-inflammatory drugs within 3 days (119 [11.0%] vs 56 [5.0%]; *P* < .001) or 7 days (134 [12.4%] vs 72 [6.4%]; *P* < .001) after randomization. No significant differences were noted between the 2 groups in terms of the other nephrotoxins, including contrast, aminoglycoside, vancomycin, chemotherapy, and angiotensin-converting enzyme inhibitor and angiotensin receptor blocker at 3 and 7 days after randomization. Compared with the usual care group, the alert group had more AKI documentation at discharge (560 patients [49.9%] vs 296 patients [27.3%]; *P* < .001). No significant differences in LOS and hospital total costs were observed between the groups.

**Table 3.** Acute Kidney Injury (AKI) Patient Care Outcomes Stratified by Study Group

Outcome	Patients, No. (%)		Difference (95% CI)	*P* value
	Usual care group (n = 1085)	Alert group (n = 1123)		
Diagnostic and therapeutic interventions				
New intravenous fluids within 2 d after randomization	670 (61.8)	927 (82.6)	20.8 (17.1 to 24.4)	<.001
Urinalysis within 2 d after randomization	141 (13.0)	407 (36.2)	23.3 (19.8 to 26.7)	<.001
Fluid intake and output measurements within 2 d after randomization	743 (68.5)	995 (88.6)	20.1 (16.8 to 23.4)	<.001
Subsequent creatinine measurement within 2 d after randomization	814 (75.0)	1039 (92.5)	17.5 (14.5 to 20.5)	<.001
Kidney ultrasonography within 2 d after randomization	6 (0.6)	59 (5.3)	4.7 (3.4 to 6.2)	<.001
Correcting anemia by reaching hemoglobin level >9.0 g/dL within 2 d after randomization	252 (23.2)	517 (46.0)	22.8 (18.9 to 26.6)	<.001
Nephrologist consultation with inpatient	222 (20.5)	263 (23.4)	2.9 (−0.5 to 6.4)	.09
Medical ICU	71 (27.6)	71 (28.6)	1.0 (−6.8 to 8.8)	.80
Medical ward	56 (23.8)	49 (21.9)	−1.9 (−9.6 to 5.8)	.62
Surgical ICU	69 (18.2)	110 (24.3)	6.1 (0.5 to 11.6)	.03
Surgical ward	26 (12.2)	33 (16.7)	4.5 (−2.4 to 11.4)	.20
Nephrotoxin exposure				
Contrast within 3 d after randomization	33 (3.0)	32 (2.8)	−0.2 (−1.7 to 1.2)	.79
Contrast within 7 d after randomization	49 (4.5)	53 (4.7)	0.2 (−1.6 to 2.0)	.82
Aminoglycoside within 3 d after randomization	5 (0.5)	4 (0.4)	−0.1 (−0.8 to 0.5)	.75
Aminoglycoside within 7 d after randomization	8 (0.7)	7 (0.6)	−0.1 (−0.9 to 0.6)	.80
Vancomycin within 3 d after randomization	26 (2.4)	26 (2.3)	−0.1 (−1.4 to 1.2)	.90
Vancomycin within 7 d after randomization	36 (3.3)	31 (2.8)	−0.5 (−2.0 to 0.9)	.45
Chemotherapy within 3 d after randomization	17 (1.6)	17 (1.5)	−0.1 (−1.1 to 1.0)	.91
Chemotherapy within 7 d after randomization	22 (2.0)	24 (2.1)	0.1 (−1.1 to 1.3)	.86
NSAID within 3 d after randomization	119 (11.0)	56 (5.0)	−6.0 (−8.3 to 3.7)	<.001
NSAID within 7 d after randomization	134 (12.4)	72 (6.4)	−6.0 (−8.4 to 3.5)	<.001
ACEI and ARB within 3 d after randomization	47 (4.3)	43 (3.8)	−0.5 (−2.2 to 1.2)	.55
ACEI and ARB within 7 d after randomization	55 (5.1)	50 (4.5)	−0.6 (−2.4 to 1.2)	.50
Administrative				
AKI documentation in the discharge record	296 (27.3)	560 (49.9)	22.6 (18.6 to 26.5)	<.001
Length of stay, median (IQR), d	19 (11-29)	20 (12-29)	1.0 (0.0 to 2.0)	.20
Hospital total costs, median (IQR), US$	21 147 (8604-38 680)	22 784 (8781-42 591)	1637 (−290 to 3632)	.09

Abbreviations: ACEI, angiotensin-converting enzyme inhibitor; ARB, angiotensin receptor blocker; ICU, intensive care unit; NSAID, nonsteroidal anti-inflammatory drug.

SI conversion factor: To convert hemoglobin to gram per liter, multiply by 10.0.

### Post Hoc Analyses

**Figure 2.**
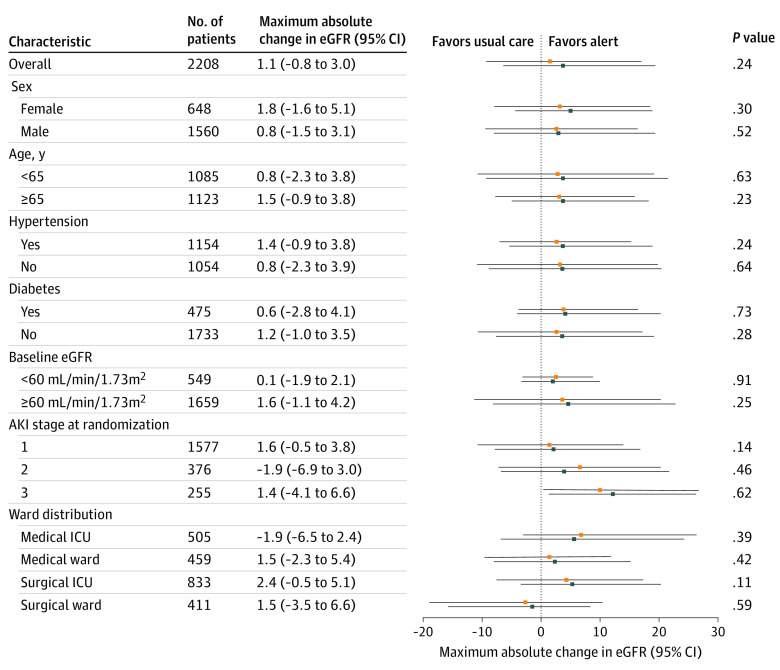
Exploratory Subgroup Analyses on Maximum Absolute Change in Estimated Glomerular Filtration Rate (eGFR) Error bars represent 95% CIs. AKI indicates acute kidney injury; ICU, intensive care unit; blue squares, AKI alert group; orange squares, usual care.

Post hoc exploratory subgroup analysis showed no significant differences between the alert group and the usual care group in the primary outcome (Figure 2; eTable 1 in Supplement 2) and in-hospital death (eTable 2 in Supplement 2). However, patients with baseline eGFR of 60 mL/min/1.73 m^2^ or higher (odds ratio [OR], 1.37; 95% CI, 1.05-1.78; *P* = .02) or patients in the surgical ICU (OR, 1.54; 95% CI, 1.08-2.20; *P* = .02) had a significantly higher risk of in-hospital dialysis in the alert group compared with the usual care group (eTable 3 in Supplement 2). There was no difference in median hospital total cost, LOS, and nephrologist consultation between the alert group and the usual care group according to the AKI stage at randomization (eTable 4 in Supplement 2).

## Discussion

To our knowledge, this RCT was the largest study conducted in the Chinese Han population to evaluate the effect of the AKI alert combined with a care bundle on the care and clinical outcomes of hospitalized patients. The results showed that the AKI alert increased the proportion of patients receiving multiple interventions and substantially increased the diagnostic rate of AKI. However, these interventions did not appear to improve kidney function and patient outcomes.

Previous research on the effect of AKI alerts on kidney function has yielded inconsistent results. A prospective study found that the alert group had more patients with a high SCr level returning to baseline level within 8 hours after AKI occurred.^5^ A quality improvement study found that the introduction of an alert system reduced the incidence of severe AKI and increased AKI recovery rates.^6^ A multicenter, sequential period analysis found a substantial decrease in the need for dialysis after the introduction of a clinical decision support system.^9^ However, the 2015 RCT by Wilson et al^13^ found no difference in SCr levels or dialysis rates between the alert and usual care groups 7 days after randomization. A 2021 RCT also did not demonstrate any improvement in dialysis rates with use of AKI alerts.^14^ Consistent with these RCTs, the present trial did not find evidence of improved short-term kidney function. Despite variations in study populations, alert methods, and intervention measures, current evidence does not support the idea that alert system implementation enhances kidney function.

This trial found that the AKI alert changed AKI interventions, aligning with earlier research findings that electronic AKI alerts were associated with increased fluid resuscitation, discontinuation of nephrotoxic medications, optimization of hemodynamic parameters, and timely nephrologist consultations.^15,18,19,20,21^ Theoretically, changes in these care behaviors have the potential to improve patient outcomes. However, this trial found that the AKI alert did not improve patient outcomes but increased the need for dialysis in surgical ICUs, which was consistent with the study findings of Wilson et al.^13^ We observed an increase in fluid resuscitation, which may contribute to an elevated dialysis rate due to excessive fluid.^22,23^ Furthermore, patients in the ICU are usually critically ill, and multiple other comorbidities may provide indications for dialysis treatment. During the era of this research, there was also controversy regarding the timing of initiating dialysis for patients with severe AKI.^24,25,26^ Exploratory subgroup analysis revealed higher dialysis rates in patients with an eGFR of at least 60 mL/min/1.73 m^2^ in the alert group. Clinicians might monitor patients with underlying kidney disease and apply more appropriate interventions, and caution is required to prevent excessive interventions for patients with better eGFR after receiving an AKI alert.

Results of this study do not support the AKI alert, and even oppose it in some cases, but it does not mean that the alert has no further research value. The contribution of the alert system to AKI epidemiological research is undeniable.^8,27^ Furthermore, research on alerts allows us to reconsider care processes for AKI, and future improvements in interventions are expected to improve the prognosis of AKI. More recent studies showed the benefits associated with the AKI alert, such as decreased 30-day mortality rate,^28^ early recovery from AKI after major surgery,^29^ and shorter LOS.^11^ The cost-effectiveness of AKI alerts also needs further research.^30^ Additionally, it is worth discussing how to identify patients who may benefit from warnings given that populations needing alerts are highly heterogeneous.^29,31,32,33^ The application of AKI alerts in pregnant individuals,^34^ kidney transplant recipients,^35^ and coronary angiography populations^36^ was explored in previous studies. In the future, by refining alert timing, content, methods, and interventions and AKI management, it is probable that AKI alert may still have a beneficial effect on the outcomes of patients.

Kidney function is not stable during AKI, and random errors resulting from this instability may bias the study results toward the null hypothesis. Therefore, we used the maximum difference in eGFR to mitigate this issue as much as possible. Since eGFR may not account for non-GFR factors, such as acute tubular necrosis or other tubular dysfunction–induced AKI, we also analyzed the maximum change in SCr levels within 7 days after randomization, which showed no significant difference between the alert and usual care groups, consistent with the results of the study by Wilson et al.^13^

### Limitations

This study has several limitations. First, as the trial was conducted at a single center, the findings may be affected by the characteristics of the population and the hospital and may not be universally applicable. Second, the care bundle included was general, nonindividualized, and nonmandatory, which may weaken the effect of the AKI alert. Third, although some measures have been taken, they may not be enough to avoid warning fatigue. Fourth, since most patients do not record urine output during hospitalization, the KDIGO creatinine standard was used to design AKI alerts, which may underestimate the actual incidence of AKI. Fifth, given the lack of universally acknowledged markers of kidney function in AKI, to eliminate the errors in assessing kidney function as much as possible, we used the maximum change in eGFR or SCr levels within 7 days after randomization and secondary outcomes.

## Conclusions

In this RCT involving patients with hospital-acquired AKI, we found that AKI alerts could change the care of AKI, but these changes did not improve short-term kidney function and other clinical outcomes. These findings suggest that a combination of high-quality interventions and AKI alerts is warranted in future clinical practice.

## Notes

Supplement 1.
Trial Protocol

Supplement 2. **eTable 1.** Subgroup Analyses on Maximum Relative Change in eGFR Based on Clinical Indices **eTable 2.** Odds Ratios of In-Hospital Death in Acute Kidney Injury Alert Group Compared With Usual Care Group **eTable 3.** Odds Ratios of In-Hospital Dialysis in Acute Kidney Injury Alert Group Compared With Usual Care Group **eTable 4.** Subgroup Analyses of Secondary Outcomes Based on AKI Stage at Randomization

Supplement 3.
Data Sharing Statement
