# Supplementary material for: Automated Electronic Alert for the Care and Outcomes of Adults With Acute Kidney Injury: A Randomized Clinical Trial
Source: JAMA Netw Open. 2024 Jan 19;7(1):e2351710. doi: 10.1001/jamanetworkopen.2023.51710 (PMC10799260; doi:10.1001/jamanetworkopen.2023.51710)
Supplement: Supplement 1. — Trial Protocol [file jamanetwopen-e2351710-s001.pdf]

STUDY PROTOCOL

Title

The Impact of Automated Electronic Alert for Acute Kidney Injury on the Outcomes of Hospitalized Patients: A single-center randomized controlled observational study

Type of research: Single-center, double blind, parallel assignment randomized controlled trial

Department in charge: Department of Nephrology

Principal Investigator: Huijuan Mao, MD, PhD

Contact number: 86-13851746819

Email: maohuijuan72@hotmail.com

Research Period: August 1, 2019 - December 31, 2021

Version number: V2.0

Version date: June 08, 2019

Abstract

**Project name**	The Impact of Automated Electronic Alert for Acute Kidney Injury on the Outcomes of Hospitalized Patients: A single-center randomized controlled observational study
**Research purposes**	Observing the impact of acute kidney injury alert on patient prognosis
**Research design**	Randomized controlled study
**Total number of cases**	3000 participants
**Participants selection**	Inclusion criteria: Patients must meet all of the following criteria:<br>Inpatient adults aged ≥ 18 years with AKI, defined according to the Kidney Disease: Improving Global Outcomes (KDIGO) creatinine criteria.
	Exclusion criteria: Any one of the following will not be included in this study:<br>1) Baseline estimated glomerular filtration rate (eGFR) lower than 15 ml/min/1.73m^2.<br>2) Admission diagnosed with end-stage kidney disease defined by the international classification of disease coding 9 (ICD-9) and ICD-10.<br>3) History of kidney transplantation.<br>4) AKI occurring outside the hospital.
**Treatment programs**	AKI alert: An AKI alert will send to the doctor in charge. The team of nephrologists would give suggestions if the doctor in charge need a renal consultation.<br>Usual care: Patients will receive standard clinical care by the doctor in charge.

**Efficacy evaluation**	Effectiveness evaluation (primary and secondary outcomes)<br>Primary outcome: the change value of eGFR after 7 days of random grouping; Estimated glomerular filtration rate changed within 7 days (Time Frame: within 7 days diagnosed with AKI)<br>Secondary outcomes: seven-day mortality, 28-day mortality, 90-day mortality, 7-day dialysis, 28-day dialysis, 90-day dialysis, the rate of stage 2 AKI, the rate of stage 3 AKI, the rate of AKI recovery at discharge, the rate of AKI recovery at 90-day, the rate of timely-recognition of AKI, the interventions for AKI.
	Safety Evaluation<br>none
**Statistical methods**	Data were expressed as mean ± standard deviation (normal distribution) or median and quartile (non-normal distribution), and t-test or rank test was used for comparison between groups; categorical data were expressed by rate, and chi-square test was used for comparison between groups. Kaplan-Meier (KM) survival curve was used for survival data, and Log-rank test was used for comparison between groups.
**Study period**	2019-08 to 2021-12

Research Background

Acute kidney injury (AKI) is an important disease burden worldwide [1-2]. About 13.3 million people worldwide suffer from AKI every year, 85% of whom live in developing countries; in addition, AKI causes 1.7 million deaths every year [3]. The incidence of AKI in the hospital in developed countries is about 10%, and the incidence of AKI in China is about 1-9% [4-5]. AKI not only greatly increases the risk of short-term death (2-3 times), but also greatly increases the risk of long-term renal dysfunction and cardiovascular and cerebrovascular events [2].

However, the timely diagnosis of AKI (diagnosed within 3 days) is not optimistic. At present, the diagnosis of AKI often relies on the changes of serum creatinine and the observation of urine output in a short period of time. However, in the early stage of AKI, there are often no obvious specific symptoms, so it is easy to miss the diagnosis. It is reported that the diagnostic registration rate of AKI in foreign countries is less than 50%, while the timely diagnosis rate of AKI in China is only about 30% [4]. More importantly, at present, the creatinine value is only detected once during the hospitalization of most patients, so there may be a large number of missed diagnoses. Missed diagnosis will significantly increase the mortality of patients, delay the opportunity to prevent the deterioration of renal function.

In view of this, the International Society of Nephrology proposed the "0by25" initiative in 2013, with the goal of timely diagnosis and treatment of potentially reversible AKI, and reducing the preventable death of AKI to zero by 2025 [6]. Our research group also investigated the incidence of AKI in hospitals in 2014, and the results showed that the incidence of AKI in hospitals was 1.6%, but only 44% were diagnosed in time, and the 30-day mortality rate was 35.3% [7]. Therefore, it is urgent to improve the timely diagnosis of AKI!

In addition to increasing clinicians' awareness of AKI, the international community is currently paying special attention to the establishment of an electronic alert system for early diagnosis of AKI [8]. The AKI alert system is a program designed to automatically identify emerging serum creatinine values in the electronic medical record system. When the test system shows that the serum creatinine value increases by 50% or more than 26.5μmol/L compared with the patient's baseline (and the uremia patient is excluded), there will be an early warning of AKI to help doctors diagnose and treat early (see the figure below for the principle). However, whether the electronic alert system can improve the prognosis of patients with AKI has not yet reached a consensus [9].

Based on the current status of AKI diagnosis at home and abroad, we have applied to the hospital and approved the proposal to establish a hospital AKI alert system. In April 2018, the AKI alert system has been tested, and currently ready to send text messages to clinicians. Therefore, we plan to design this randomized controlled study to observe the impact of acute kidney injury alert on patient care and prognosis.

We speculate that the early warning system for acute kidney injury in hospitals is expected to significantly improve the rate of timely diagnosis of acute kidney injury, reduce the use of nephrotoxic drugs, take early intervention measures such as blood volume supplementation, reduce the degree of kidney injury, and benefit the majority of hospitalized patients. If it is proven to be effective, it can be extended to large hospitals in the province and even the country, and ultimately significantly improve the quality of AKI diagnosis and treatment and reduce the burden of AKI in our country!

Study objective

Main objective: To observe whether the AKI early warning system can improve the medium and short-term human/renal survival of patients.

Secondary objective: To observe the impact of AKI early warning system on AKI intervention measures.

Study Design, Participants and Interventions

1. Study Design

1.1 Study design: single-center, double blind, parallel-group randomized controlled trial

1.2 Study participants

a. Screening criteria: Patients must meet all of the following conditions:

1) Chinese male or female over 18 years old;

2) AKI newly diagnosed by the AKI alert system;

3) There were no diagnoses of "uremia", "chronic kidney disease G 5 ", and "kidney transplantation" in the admission diagnosis.

Table 1. Creatinine criteria for diagnosis of KDIGO AKI

Stages	serum creatinine standard
1	Creatinine increased by > 0.3mg/dl within 2 days or increased by more than

	1.5-1.9 times within 7 days
2	2.0-2.9 times the baseline value within 7 days
3	3.0 times the baseline value within 7 days

b. Exclusion criteria: Any one of the following will not be included in this study:

1) Patients with known chronic kidney disease G5 (eGFR<15ml/min/1.73m2) or kidney transplantation;

2) AKI has occurred before the patient's alert of AKI;

c. Rejection criteria

The patient dies unexpectedly, such as in a traffic accident. Reasons: Considering that the cause of death of the patient has little to do with treatment.

1.3 grouping

Eligible AKI patients were randomly divided into AKI alert group and usual care group. In the alert group, the AKI alert system gave early warning; in the usual care group, the AKI alert system did not give early warning.

1.4 Randomization

Random numbers were generated according to seed number 20180101, and patients were divided into 4 blocks according to surgical/internal medicine wards and ICU /non-ICU. Each block was divided into 2 groups on average.

1.5 Intervention measures: AKI alert

AKI alert is based on the KDIGO serum creatinine diagnostic criteria of AKI to determine whether the patient is AKI. When AKI is diagnosed, the hospital information system will send a text message to the physician in charge , which reads : "[Jiangsu Provincial People's Hospital] Friendly Reminder: Hello doctor, the patient in bed **, in the ** ward, admitted on **, has a serum creatinine result of ** from the ** test time. Based on the creatinine result, acute kidney injury is probable to occur. Acute kidney injury requires optimization of hemodynamics, discontinuation of unnecessary nephrotoxic drugs, and adjustment of antimicrobial drug dosage and dialysis if necessary. Please be vigilant and handle it accordingly. Thank you! For diagnosis and treatment inquiries, please contact the nephrology consultation service at the kidney consultation phone number**". If a patient experiences multiple episodes of AKI, the messages

will only be sent up to a maximum of 3 times to prevent warning fatigue among clinicians and researchers.

1.6 Research endpoints

1) The main research endpoint: the change value of eGFR 7 days after randomization (the eGFR value minus the creatinine peak corresponds to the eGFR at the time of early warning of AKI; if the patient dies, it is considered that all renal function has been lost);

Note: eGFR is calculated using the creatinine formula of CKD-EPI.

2) Secondary research endpoints: as shown in Table 2

Table 2. Secondary Study Endpoints

Endpoints and definitions	Data Sources
Mortality rate	
7-day mortality	medical records
Discharge mortality	medical records
28-day mortality	Follow up
90-day mortality	Follow up
Dialysis rate	
7-day dialysis rate	medical records
Discharge dialysis rate	medical records
28-day dialysis rate	Follow up
90-day dialysis rate	Follow up
Renal Failure	
Proportion of progression to AKI stage 2	medical records
Proportion of progression to AKI stage 3	medical records
Renal function recovery (90 days)	
Full recovery	Follow up
Partial recovery	Follow up
No recovery	Follow up
Hospital Duration and Costs	
Hospital stays	medical records
ICU time	medical records
Hospital costs	medical records
Diagnosis	
AKI diagnosis rate	medical records
Intervention	
Contrast agent use after AKI	Doctor order system
Liquid input	Doctor order system
Aminoglycoside use	Doctor order system
NSAIDs use _	Doctor order system
ACEI/ARB drugs	Doctor order system

Consult a nephrologist	Doctor order system
Urinalysis	Doctor order system
Kidney ultrasound	Doctor order system

1.7 Collect data

The observation points of this study included admission, when AKI occurred (i.e. when randomized), 7 days after AKI occurred, discharge and 90 days after AKI.

Node 1: On admission. Observation items include 1) demographic data, including gender, age, race, height, weight, BMI, and household registration; 2) patients' main diagnosis, hospitalization department, past medical history, and Charlson comorbidity score;

Node 2: When AKI occurs. Observation items AKI occurrence time, APACHE II score, SOFA score, blood routine on the day of AKI occurrence or last time, liver function, renal function, medication during hospitalization, use of contrast medium, surgical conditions, number of extrarenal organ failure, whether renal replacement therapy was performed;

Node 3: 7 days after AKI occurred. Observation items include changes in renal function, AKI recovery, and whether renal replacement therapy is performed;

Node 4: When discharged from the hospital. The observation items included discharge time, hospitalization time, hospitalization expenses, in-hospital death/dialysis, and renal function recovery after discharge.

Node 5: AKI 28 days. Observation items include AKI 28-day death/dialysis and 28-day renal function recovery.

Node 6: AKI 90 days. Observation items include AKI 90-day death/dialysis and 90-day renal function recovery.

1.8 Sample size:

From August 2019 to December 2021, 3000 patients are planned to be included, with 1500 cases in the test group and 1500 cases in the control group.

Calculation reason: On the basis of our pilot data, we estimated a SD of 13.4 ml/min/1.73m^2 of absolute eGFR change averaged across groups. We considered a relative 10% reduction in the primary outcome (19.0 to 17.1 ml/min/1.73m^2) was clinically significant. At a 2-tailed 0.025 level test for an overall significance level of $\alpha = 0.05$, a sample size of 1050 in each group of the study achieves 90% power to detect the difference. Giving the potential for the 30% of

patients without last creatinine, the sample size was inflated to 3000 cases.

Study flow

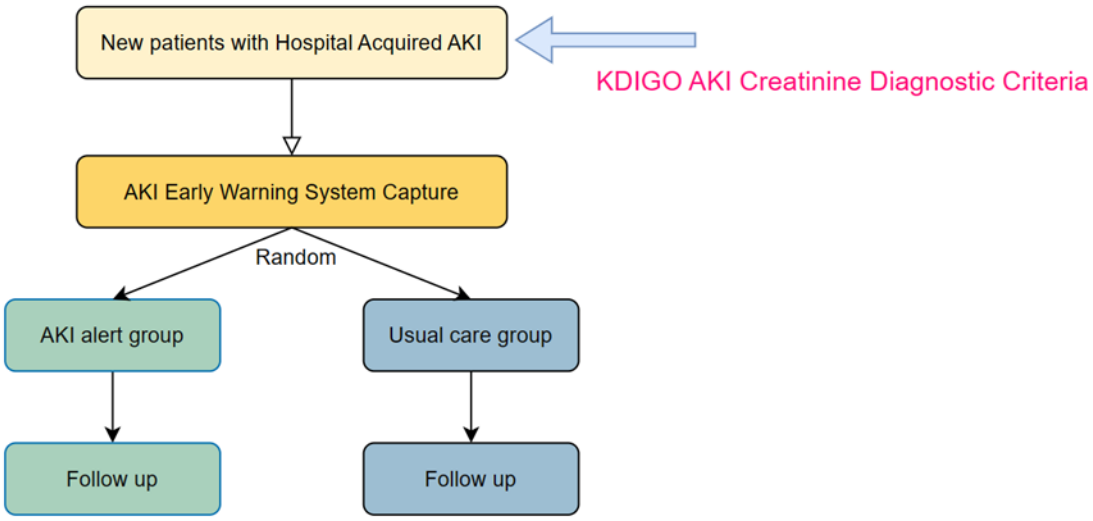

Study flow chart

The name and specification of the drug used in the study

none.

Combined medication

none.

Observation of adverse events

none.

Research Quality Control and Quality Assurance

The research meeting will strictly follow the research plan and strictly abide by the collection point time. Nothing special.

Data security monitoring

Clinical research will formulate corresponding data safety monitoring plans according to the degree of risk. All adverse events are recorded in detail, properly handled and tracked until they are properly resolved or the condition is stable, and timely report serious adverse events and unexpected events to the ethics committee, competent authorities, sponsors, and drug regulatory authorities in accordance with regulations; the main investigator conducts a cumulative review of all adverse events on a regular basis, and holds investigator meetings to evaluate the risks

and benefits of the research when necessary; double-blind trials can be unblinded urgently when necessary to ensure the safety and rights of subjects; research with greater than the minimum risk will arrange independent data monitors to monitor the research data, and high-risk research will establish independent data safety monitoring The committee monitors the accumulated safety and efficacy data to make a recommendation on whether to proceed with the study (This paragraph does not fit our research).

Statistical processing

SAS9.2 was used for statistical analysis. Measurement data were expressed as mean ± standard deviation (normal distribution) or median and quartile (non-normal distribution), and t-test or rank test was used for comparison between groups; categorical data were expressed by rate, and chi-square test was used for comparison between groups. Kaplan-Meier (KM) survival curve was used for survival data, and Log-rank test was used for comparison between groups.

Ethics of Clinical Research

Clinical research will follow relevant regulations such as the Declaration of Helsinki of the World Medical Assembly. The clinical study will only be conducted after the ethical committee approves the trial protocol. This study is an observational study, and a written informed consent free application is made to the Ethics Committee. The research process will protect the personal privacy and data confidentiality of the subjects.

References

1. Kidney Disease: Improving Global Outcomes (KDIGO) Acute Kidney Injury Work Group. KDIGO clinical practice guideline for Acute kidney injury. Kidney International Supplements. 2012;2(1):1–138.
2. Lewington AJ, Cerda J, Mehta RL. Raising awareness of acute kidney injury: a global perspective of a silent killer. Kidney Int. 2013;84(3):457–67.
3. Susantitaphong P, Cruz DN, Cerda J, Abulfaraj M, Alqahtani F, Koulouridis I, Jaber BL, Acute Kidney Injury Advisory Group of the American Society of N. World incidence of AKI: a meta-analysis. Clin J Am Soc Nephrol. 2013;8(9):1482–93.
4. Yang L, Xing G, Wang L, Wu Y, Li S, Xu G, He Q, Chen J, Chen M, Liu X, et al. Acute kidney injury in China: a cross-sectional survey. Lancet. 2015;386(10002):1465–71.
5. Xu X, Nie S, Liu Z, et al. Epidemiology and Clinical Correlates of AKI in Chinese Hospitalized Adults. Clinical journal of the American Society of Nephrology : CJASN 2015; 10(9): 1510-8.
6. Mehta RL, Cerdá J, Burdmann EA, et al. International Society of Nephrology's 0by25

initiative for acute kidney injury (zero preventable deaths by 2025): a human rights case for nephrology. Lancet. 2015 Jun 27;385(9987):2616-43.

7. Cheng X, Wu B, Liu Y, et al. Incidence and diagnosis of Acute kidney injury in hospitalized adult patients: a retrospective observational study in a tertiary teaching Hospital in Southeast China. BMC Nephrol. 2017 Jun 24;18(1):203.
8. Palevsky PM. Electronic Alerts for Acute Kidney Injury. Am J Kidney Dis. 2018 Jan;71(1):1-2.
9. Lachance P, Villeneuve PM, Rewa OG, et al. Association between e-alert implementation for detection of acute kidney injury and outcomes: a systematic review. Nephrol Dial Transplant. 2017 Feb 1;32(2):265-272.
