# Supplementary material for: Automated Electronic Alert for the Care and Outcomes of Adults With Acute Kidney Injury: A Randomized Clinical Trial
Source: JAMA Netw Open. 2024 Jan 19;7(1):e2351710. doi: 10.1001/jamanetworkopen.2023.51710 (PMC10799260; doi:10.1001/jamanetworkopen.2023.51710)
Supplement: Supplement 2. — eTable 1. Subgroup Analyses on Maximum Relative Change in eGFR Based on Clinical Indices eTable 2. Odds Ratios of In-Hospital Death in Acute Kidney Injury Alert Group Compared With Usual Care Group eTable 3. Odds Ratios of In-Hospital Dialysis in Acute Kidney Injury Alert Group Compared With Usual Care Group eTable 4. Subgroup Analyses of Secondary Outcomes Based on AKI Stage at Randomization [file jamanetwopen-e2351710-s002.pdf]

Supplemental Online Content

Li T, Wu B, Li L, et al. Automated electronic alert for the care and outcomes of adults with acute kidney injury: a randomized clinical trial. *JAMA Netw Open*. 2024;7(1):e2351710. doi:10.1001/jamanetworkopen.2023.51710

eTable 1. Subgroup Analyses on Maximum Relative Change in eGFR Based on Clinical Indices

eTable 2. Odds Ratios of In-Hospital Death in Acute Kidney Injury Alert Group Compared With Usual Care Group

eTable 3. Odds Ratios of In-Hospital Dialysis in Acute Kidney Injury Alert Group Compared With Usual Care Group

eTable 4. Subgroup Analyses of Secondary Outcomes Based on AKI Stage at Randomization

This supplemental material has been provided by the authors to give readers additional information about their work.

© 2024 Li T et al. *JAMA Network Open*

eTable 1. Subgroup analyses on maximum relative change in eGFR based on clinical indices

**Maximum relative change in eGFR (%)**	Usual care (n = 1085)	Alert (n = 1123)	p value
**All patients**	8 (-19, 44)	9 (-15, 47)	0.255
**Sex**			
Female (n = 648)	12 (-20, 48)	16 (-10, 53)	0.202
Male (n = 1560)	6 (-18, 42)	8 (-17, 45)	0.684
**Age**			
< 65 (n = 1085)	6 (-20, 45)	8 (-18, 46)	0.669
⩾ 65 (n =1123)	10 (-17, 43)	11 (-11, 48)	0.217
**Hypertension**			
Yes (n = 1154)	8 (-16, 43)	9 (-12, 47)	0.349
No (n = 1054)	8 (-22, 47)	9 (-18, 47)	0.480
**Diabetes**			
Yes (n = 475)	13 (-10, 42)	11 (-9, 48)	0.760
No (n = 1733)	6 (-21, 44)	9 (-16, 47)	0.302
**Baseline eGFR**			
< 60 (n = 549)	10 (-12, 35)	8 (-12, 39)	0.988
⩾ 60 (n = 1659)	6 (-21, 47)	10 (-15, 49)	0.217
**AKI stage at randomization**			
1 (n = 1577)	3 (-20, 33)	5 (-16, 39)	0.206
2 (n = 376)	21 (-22, 52)	13 (-22, 53)	0.562
3 (n = 255)	42 (1, 78)	45 (3, 100)	0.415
**Ward**			
Medical intensive care unit (n = 505)	21 (-9, 69)	17 (-15, 100)	0.517
Medical ward (n = 459)	4 (-25, 41)	6 (-19, 43)	0.401
Surgical intensive care unit (n = 833)	10 (-14, 41)	12 (-7, 45)	0.103
Surgical ward (n = 411)	-8 (-38, 26)	-4 (-32, 30)	0.612

Abbreviations: eGFR, estimated glomerular filtration rate; AKI, acute kidney injury

© 2024 Li T et al. *JAMA Network Open*

eTable 2. Odds ratios of in-hospital death in acute kidney injury alert group compared with usual care group

In-hospital death	Events, n (%)		Odds ratio (95% CI)	p value	p for interaction
	Usual care (n = 1085)	Alert (n = 1123)			
**All patients**	192 (17.7%)	223 (19.9%)	1.15 (0.93-1.43)	0.194	
**Sex**					
Female (n = 648)	63 (9.7%)	72 (11.1%)	0.99 (0.68 – 1.45)	0.955	0.359
Male (n = 1560)	129 (8.3%)	151 (9.7%)	1.23 (0.95 - 1.59)	0.122	
**Age**					
< 65 (n = 1085)	73 (6.7%)	95 (8.8%)	1.24 (0.89 – 1.72)	0.207	0.640
⩾ 65 (n =1123)	119 (10.6%)	128 (11.4%)	1.12 (0.84 – 1.48)	0.448	
**Hypertension**					
Yes (n = 1154)	100 (8.7%)	108 (9.4%)	1.06 (0.78 - 1.43)	0.716	0.425
No (n = 1054)	92 (8.7%)	115 (10.9%)	1.26(0.93 – 1.71)	0.140	
**Diabetes**					
Yes (n = 475)	41 (8.6%)	56 (11.8%)	1.22 (0.78 – 1.91)	0.396	0.779
No (n = 1733)	151 (8.7%)	167 (9.6%)	1.13 (0.88 – 1.44)	0.326	
**Baseline eGFR**					
⩾ 60 (n = 1659)	141 (8.5%)	171 (10.3%)	1.19 (0.93 – 1.52)	0.176	0.648
< 60 (n = 549)	51 (9.3%)	52 (9.5%)	1.06 (0.69 – 1.62)	0.968	
**AKI stage at randomization**					0.741
1 (n = 1577)	114 (7.2%)	129 (8.2%)	1.16 (0.88 - 1.52)	0.301	
2 (n = 376)	46 (12.2%)	49 (13.0%)	0.90 (0.57 – 1.44)	0.671	
3 (n = 255)	32 (12.6%)	45 (17.6%)	1.44 (0.84 - 2.47)	0.187	
**Ward distribution**					0.674
Medical ICU (n = 505)	192 (18%)	223 (20%)	1.15 (0.93-1.43)	0.194	
Medical ward (n = 459)	33 (14%)	36 (16%)	1.17 (0.70-1.96)	0.543	
Surgical ICU (n = 833)	48 (13%)	72 (16%)	1.307 (0.882-1.938)	0.183	
Surgical ward (n = 411)	26 (12%)	23 (12%)	0.945 (0.520-1.718)	0.854	

© 2024 Li T et al. *JAMA Network Open*

Abbreviations: eGFR, estimated glomerular filtration rate; AKI, acute kidney injury; ICU, intensive care unit.

© 2024 Li T et al. *JAMA Network Open*

eTable 3. Odds ratios of in-hospital dialysis in acute kidney injury alert group compared with usual care group

In-hospital dialysis	Events, n (%)		Odds ratio (95% CI)	p value	p for interaction
	Usual care (n = 1085)	Alert (n = 1123)			
**All patients**	172 (15.8%)	211 (17.1%)	1.228 (0.984 - 1.532)	0.069	
**Sex**					0.534
Female (n = 648)	47 (7.2%)	70 (10.8%)	1.366 (0.909 - 2.052)	0.133	
Male (n = 1560)	125 (8.0%)	141 (9.0%)	1.171 (0.899 - 1.525)	0.243	
**Age**					0.801
< 65 (n = 1085)	87 (8.0%)	114 (10.5%)	1.258 (0.924 - 1.713)	0.145	
⩾ 65 (n =1123)	85 (7.6%)	97 (8.6%)	1.188 (0.865 - 1.633)	0.288	
**Hypertension**					0.703
Yes (n = 1154)	86 (7.4%)	109 (9.4%)	1.281 (0.94 - 1.746)	0.117	
No (n = 1054)	86 (8.2%)	102 (9.7%)	1.175 (0.856 - 1.612)	0.317	
**Diabetes**					0.202
Yes (n = 475)	31 (6.5%)	54 (11.4%)	1.621 (0.999 - 2.631)	0.051	
No (n = 1733)	141 (8.1%)	157 (9.1%)	1.137 (0.886 - 1.46)	0.314	
**Baseline eGFR**					0.194
⩾ 60 (n = 1659)	110 (6.6%)	151 (9.1%)	1.365 (1.045 - 1.783)	0.022	
< 60 (n = 549)	62 (11.3%)	60 (10.9%)	0.991 (0.662 - 1.482)	0.964	
**AKI stage at randomization**					0.511
1 (n = 1577)	94 (6.0%)	104 (6.6%)	1.121 (0.832 - 1.51)	0.453	
2 (n = 376)	31 (8.2%)	47 (12.5%)	1.418 (0.854 - 2.354)	0.177	
3 (n = 255)	47 (18.4%)	60 (23.5%)	1.312 (0.796 - 2.162)	0.287	
**Ward distribution**					0.066
Medical intensive care unit (n = 505)	64 (25%)	61 (25%)	0.984 (0.657-1.474)	0.937	
Medical ward (n = 459)	28 (12%)	23 (10%)	0.846 (0.471-1.518)	0.575	
Surgical intensive care unit (n = 833)	59 (16%)	100 (22%)	1.541 (1.080-2.199)	0.017	
Surgical ward (n = 411)	21 (10%)	27 (14%)	1.444 (0.787-2.647)	0.235	

Abbreviations: eGFR, estimated glomerular filtration rate; AKI, acute kidney injury

© 2024 Li T et al. *JAMA Network Open*

eTable 4. Subgroup analyses of secondary outcomes based on AKI stage at randomization

	Usual care (n = 1085)	Alert (n = 1123)	p value
**Median hospital total cost ($)**			
**All patients**	21147 (8604-38680)	22784 (8781-42591)	0.090
**AKI stage at randomization**			
1 (n = 1577)	21615 (8373, 37629)	24005 (8913, 41360)	0.090
2 (n = 376)	19191 (9200, 36251)	22098 (9624, 44177)	0.195
3 (n = 255)	21701 (11427, 52543)	17909 (7942, 46159)	0.407
**Length of stay (days)**			
**All patients**	19 (11-29)	20 (12–29)	0.200
**AKI stage at randomization**			
1 (n = 1577)	19 (11, 28)	19 (12, 29)	0.221
2 (n = 376)	18 (9, 28)	21 (12, 30)	0.167
3 (n = 255)	22 (11, 35)	20 (10, 35)	0.366
**Kidney consults inpatient**			
**All patients**	222 (20.5%)	263 (23.4%)	0.093
**AKI stage at randomization**			
1 (n = 1577)	127 (16.1%)	135 (17.1%)	0.596
2 (n = 376)	40 (22.9%)	60 (29.9 %)	0.126
3 (n = 255)	55 (45.1%)	68 (51.1%)	0.333

Abbreviations: AKI, acute kidney injury.

© 2024 Li T et al. *JAMA Network Open*
