# Supplementary material for: Automated Electronic Alert for the Care and Outcomes of Adults With Acute Kidney Injury: A Randomized Clinical Trial
Source: JAMA Netw Open. 2024 Jan 19;7(1):e2351710. doi: 10.1001/jamanetworkopen.2023.51710 (PMC10799260; doi:10.1001/jamanetworkopen.2023.51710)
Supplement: Supplement 3. — Data Sharing Statement [file jamanetwopen-e2351710-s003.pdf]

Data Sharing Statement

Li. Automated Electronic Alert for the Care and Outcomes of Adults With Acute Kidney Injury. *JAMA Netw Open*. Published January 19, 2024. doi:10.1001/jamanetworkopen.2023.51710

Data
Data available: No
